# Targeting glycolysis: exploring a new frontier in glioblastoma therapy

**DOI:** 10.3389/fimmu.2024.1522392

**Published:** 2025-01-14

**Authors:** Lu Yang, Sijia Li, Lei Yu, Jiao Leng, Na Li

**Affiliations:** Department of Oncology, Suining Central Hospital, Suining, Sichuan, China

**Keywords:** glioblastoma, glycolytic metabolic reprogramming, lactate, tumor microenvironment, targeted therapy

## Abstract

Glioblastoma(GBM) is a highly malignant primary central nervous system tumor that poses a significant threat to patient survival due to its treatment resistance and rapid recurrence.Current treatment options, including maximal safe surgical resection, radiotherapy, and temozolomide (TMZ) chemotherapy, have limited efficacy.In recent years, the role of glycolytic metabolic reprogramming in GBM has garnered increasing attention. This review delves into the pivotal role of glycolytic metabolic reprogramming in GBM, with a particular focus on the multifaceted roles of lactate, a key metabolic product, within the tumor microenvironment (TME). Lactate has been implicated in promoting tumor cell proliferation, invasion, and immune evasion. Additionally, this review systematically analyzes potential therapeutic strategies targeting key molecules within the glycolytic pathway, such as Glucose Transporters (GLUTs), Monocarboxylate Transporters(MCTs), Hexokinase 2 (HK2), 6-Phosphofructo-2-Kinase/Fructose-2,6-Biphosphatase 3 (PFKFB3), Pyruvate Kinase Isozyme Type M2 (PKM2), and the Lactate Dehydrogenase A (LDHA). These studies provide a novel perspective for GBM treatment. Despite progress made in existing research, challenges remain, including drug penetration across the blood-brain barrier, side effects, and resistance. Future research will aim to address these challenges by improving drug delivery, minimizing side effects, and exploring combination therapies with radiotherapy, chemotherapy, and immunotherapy to develop more precise and effective personalized treatment strategies for GBM.

## 1 Introduction

Glioblastoma (GBM) is a highly malignant and prognostically poor primary brain tumor, classified as Grade IV glioma by the World Health Organization (WHO), accounting for approximately 15% of all primary intracranial malignant tumors (1, 2). GBM is characterized by its aggressive nature, high recurrence rate, and treatment resistance, resulting in a dismal prognosis with a median survival of less than 15 months despite maximal safe surgical resection, radiotherapy, and temozolomide (TMZ) chemotherapy (3, 4). The primary challenges in GBM treatment stem from its high heterogeneity and complex microenvironment. Extensive research has implicated glycolytic metabolic reprogramming as a potential initiating event in GBM development and as a regulator of the plasticity that contributes to glioma heterogeneity (5, 6). Beyond providing essential energy and biosynthetic precursors for tumor cells, glycolytic metabolic reprogramming profoundly impacts the TME (7). The shift from mitochondrial oxidative phosphorylation (OXPHOS) to aerobic glycolysis (Warburg effect) is a hallmark of tumor cell growth and metastasis. Consequently, targeting aerobic glycolysis in tumor cells holds great promise for anti-tumor therapy. Studies have shown that reversing the energy production pathway of OXPHOS can induce the differentiation of GBM cells into astrocytes (8). Therefore, interventions targeting the abnormal glycolytic characteristics of GBM have emerged as a crucial direction in modern cancer therapy research. Significant progress has been made in understanding tumor metabolic reprogramming in GBM; however, the roles of lactate, the main product of glycolysis, in tumor development remain incompletely elucidated.

This study aims to explore the critical role of glycolytic metabolic reprogramming in GBM, with a specific focus on the mechanisms of lactate’s action within the TME. By thoroughly analyzing GBM glycolysis, this study seeks to provide a theoretical basis for developing novel therapeutic targets and improving clinical outcomes for GBM patients.

## 2 The role of lactate, a glycolytic metabolic product, in shaping the GBM tumor microenvironment and immune response

**Figure 1:**
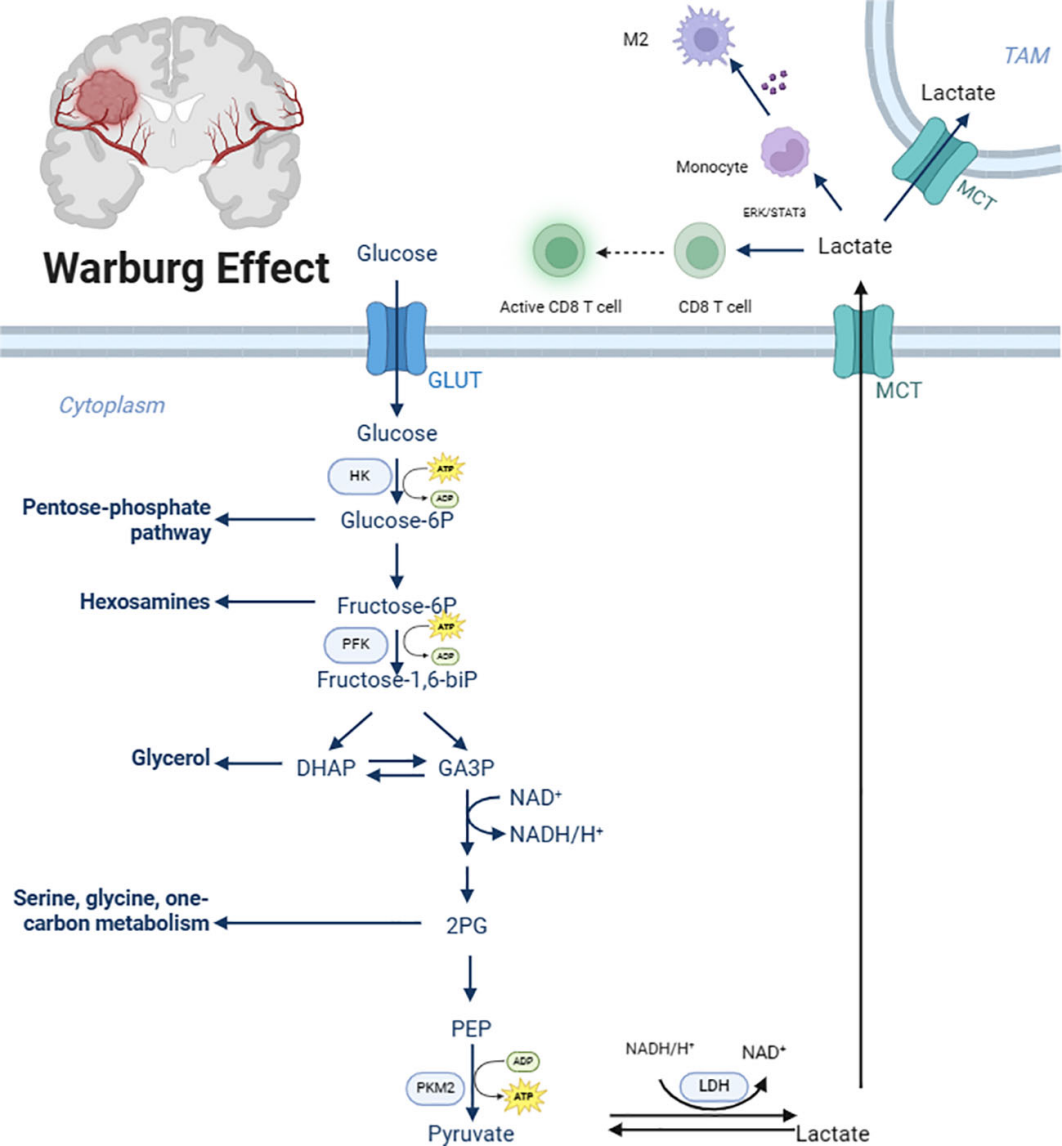
Glycolytic metabolic reprogramming in glioblastoma. GLUT, Glucose transporter; HK, Hexokinase 2; Glucose-6P, Glucose-6 phosphate; Fructose-6P, fructose-6-phosphate; PFK, Phosphofructokinase; fructose-1,6-bip, Fructose-1,6-biphosphate; GA3P, glyceraldehyde-3-phosphate; 2PG, 2-phosphoglycerate; PEP, Phosphoenolpyruvate; PKM2, Pyruvate Kinase 2; MCT, Monocarboxylate transporter. Created with BioRender.com.

In physiological conditions, cells exposed to ample oxygen typically utilize the highly efficient and energy-rich OXPHOS system for energy production, maximizing energy extraction from glucose molecules and converting it into adenosine triphosphate (ATP) for cellular use (9). However, in malignant tumors like GBM, cancer cells exhibit a pathological metabolic shift, preferentially relying on glycolysis, an inefficient energy production pathway, even in the presence of sufficient oxygen (10). Although glycolysis produces fewer ATP molecules per glucose unit compared to OXPHOS, its rapid rate allows for the swift accumulation of ATP, fulfilling the energy demands of rapidly proliferating and invasive tumor cells (11, 12). This phenomenon, first observed by Otto Warburg in the 20th century, is widely known as the Warburg effect or aerobic glycolysis and is a common metabolic feature in numerous malignancies (**Figure 1**).

### 2.1 Physiological roles of lactic acid

The excessive activation of glycolytic metabolic reprogramming plays a crucial role in GBM development, progression, and immune resistance. It not only provides a rapid means of ATP generation to meet the immense energy requirements of tumor cells but also influences the TME. The product of glycolysis, lactate, has been shown to play significant roles in GBM proliferation, differentiation, and progression (13). Lactate serves as an internal energy source for GBM tumor cells, converting within the mitochondria via the tricarboxylic acid (TCA) cycle to produce ATP, partially uncoupling energy production from glycolysis and allowing glucose to be primarily utilized for cell proliferation and metastasis. Additionally, the accumulation of lactate within the GBM TME leads to a decrease in environmental pH, creating an acidic TME (14). The acidic TME generates an inwardly directed proton gradient across the cancer cell plasma membrane, providing a driving force for proton-coupled transporters to enhance the supply of select nutrients. Research by Colen CB et al. demonstrated that inhibition of lactate efflux in rats with intracranially implanted glioma cells significantly impaired tumor invasion, potentially through the promotion of Epithelial-Mesenchymal Transition (EMT), tumor angiogenesis, metastasis, and colonization of distant organs (15–17). Besides, clinical studies have confirmed that elevated lactate levels are associated with GBM grading, higher Ki-67 index, larger tumor volume, and poorer prognosis (18, 19).

### 2.2 Regulation of lactate on the activation status and functions of macrophages

Macrophages are the most abundant immune cells within the GBM TME, comprising over 30% of infiltrating cells and reaching up to 70% in certain cases (20, 21). Studies have shown that microglia, a type of macrophage, play a crucial role in GBM progression (22). The production of lactate by cancer cells in GBM has been reported to promote M2 polarization and tumor angiogenesis of tumor-associated macrophages (TAMs) (23), the possible mechanism is that lactate can upregulate the expression of Vascular Endothelial Growth Factor (VEGF) and ARG1 genes through HIF1a-mediated mechanisms, promoting macrophage M2 polarization (24). *In vitro* experiments by Noe JT et al. revealed that supplementation of lactate to glucose-starved mouse bone marrow-derived macrophages increased histone H3 Lysine 9 (H3K9) acetylation in the ARG1 and Retnla promoter regions, indicating that lactate can regulate macrophage gene expression through epigenetic mechanisms (25). Lonhitano et al. explored the interaction between lactate and Lnsulin-like Growth Factor-binding Protein 6 (IGFBP6) in GBM cells, revealing that lactate can regulate IGFBP6 expression, further modulating microglial polarization and contributing to tumor development and therapeutic resistance (26). Similarly, Yan C et al. demonstrated that the proton-sensing G Protein-coupled Receptor (GPCRs) GPR65 can respond to lactate stimulation via the cAMP/PKA/CREB signaling pathway, triggering HMGB1 release and promoting M2 polarization of TAMs, subsequently enhancing glioma cell proliferation, migration, invasion, and mesenchymal transition (27). Li Y et al. showed that lactate depletion and SIRPα signaling blockade could potentially re-educate TAMs, reversing the immunosuppressive TME, enhancing macrophage anti-tumor activity, and effectively inhibiting tumor growth (28). Furthermore, research conducted by Ye Z et al. has shown that when macrophages in GBM exhibit high oxidative stress and reduced antigen presentation, it leads to a significant decrease in the number and function of CD8+ T cells, ultimately promoting an immunosuppressive microenvironment and the growth and progression of GBM (29)

### 2.3 Regulation of lactate on the activation status and functions of T cells

Lactate is not only a byproduct of tumor cell metabolism but also an important immunoregulatory factor that can influence the activation state and function of T cells (30), T cells are diverse and can be primarily divided into two types: CD8+ T cells and CD4+ T cells. On one hand, lactate acts as a physiological carbon source, facilitating effective CD8+ T cell activation even when glucose is abundant (31). Research has shown that lactate reduces the binding of Glyceraldehyde-3-phosphate Dehydrogenase (GAPDH) to cytokine mRNA, thereby releasing more free cytokine mRNA and enhancing cytokine production by regulatory T cells (32–34). The main mechanism involves lactate inhibiting histone deacetylase activity, increasing H3K27 acetylation in the TCF7 super enhancer region, and upregulating TCF7 gene expression, thereby enhancing CD8 T cell stemness and anti-tumor efficacy, Feng Q et al. demonstrated that lactate-pretreated CD8 T cells effectively suppressed tumor growth when transferred to tumor-bearing mice, providing evidence for the intrinsic role of lactate in anti-tumor immunity, independent of its pH-dependent effects, which may advance the development of cancer immunotherapy (35).

On the other hand, lactate-induced TME acidification inhibits CD8 T cell proliferation, cytokine production, and cytotoxic function. This effect can be reversed by adjusting the pH of lactate-containing cell culture media to 7.4, restoring the cytokine production ability of human CD8 T cells and highlighting the importance of the acidic environment for T cell function (36).

Wang Z et al. conducted an RNA-seq analysis to explore the role of lactate in the progression of GBM, uncovering that elevated lactate levels in the TME impact the migration and infiltration rate of CD8+ T cells in GBM. Specifically, in a high-lactate TME, the migratory capacity of CD8+ T cells is inhibited, resulting in decreased infiltration of these cells within the tumor tissue. This effect may be mediated through the binding of lactate to specific receptors on the surface of CD8+ T cells, such as GPR65, which in turn influences intracellular signaling pathways, including MAPK and WNT/β-catenin signaling (30).

Furthermore, lactate has been shown to regulate Foxp3-dependent RNA splicing through the Cytotoxic T-lymphocyte-Associated Protein 4 (CTLA-4) pathway, maintaining the stable phenotype and function of tumor-infiltrating regulatory T cells (Tregs) (37). The balance between Programmed Cell Death Protein 1(PD-1)-expressing CD8 T cells and Tregs within the TME determines the clinical efficacy of PD-1 blockade therapy. Kumagai S et al. demonstrated that lactate in highly glycolytic TMEs upregulates PD-1 expression on Treg cells while suppressing PD-1 expression on effector T cells, facilitating tumor cell evasion of the immune system and promoting tumor progression (38). Moreover, Fischer K’s research indicates that high extracellular lactate levels block the efflux of lactate from T cells (36). Lactate enters CD4+ T cells through MCT1 and is converted to pyruvate by LDHB, thereby driving the TCA cycle and reducing glycolysis within these cells. This metabolic shift not only hinders the proliferation of CD4+ T cells but also enhances the expansion of Tregs (39). Building on this, Zhang YT et al. further investigated the mechanisms by which lactate regulates CD4+ T cells. Their study revealed that lactate promotes the conversion of α-ketoglutarate (α-KG) to 2-hydroxyglutarate (2HG) by increasing the expression and activity of Lactate Dehydrogenase A (LDHA). This alteration affects mTOR phosphorylation and HIF-1α synthesis, ultimately leading to an increased proportion of Treg cells (40).

CD47 plays a prominent role in the immune evasion pathways of GBM and other cancers by delivering a “do not eat me” signal through binding to SIRPα expressed on macrophages and microglia (41), Similarly, research by Wang S et al. has shown that lactic acid modulates both adaptive and innate immune responses by inducing CD47 expression and STAT3 activation. Furthermore, compared to either treatment alone, combination therapy with DCA and anti-CD47 increased the infiltration of CD4+ and CD8+ T cells (42).

In summary, lactate remodels the TME through complex signaling networks, inducing an immunosuppressive environment and contributing to the establishment of resistance.

## 3 Targeting the glycolytic metabolic reprogramming pathway in GBM 

### 3.1 Targeting transporter

#### 3.1.1 Glucose transporters

The initial step in glycolysis involves the transport of glucose molecules from the extracellular environment into the cytoplasmic matrix. This crucial transport action is primarily mediated by the GLUT family members, characterized by their high affinity for glucose and efficient import of glucose into cells to sustain the survival, proliferation, and growth of cancer cells (43, 44). In GBM, the expression of GLUT-1 and GLUT-3 is particularly prominent, and their levels correlate with reduced patient survival (45). Extensive evidence suggests that GLUT1 expression is upregulated in various malignancies and is associated with poor prognosis (45, 46). Studies have demonstrated that silencing GLUT1 expression effectively reduces glioma stem cell (GSC) tumor sphere formation, decreases self-renewal and proliferation *in vitro*, indicating its potential as a therapeutic target in GBM (46). Zhang Z et al. showed that mutating the CYS207 residue of GLUT1 to serine abolished its palmitoylation and membrane localization, resulting in decreased glycolysis, reduced cancer cell proliferation, and suppression of GBM tumorigenic potential (47). The research by Y. Li et al. found that HSP90B1 is significantly upregulated in radioresistant GBM cell lines, and that HSP90B1 promotes the localization of GLUT1 on the plasma membrane, enhances glycolytic activity, and thereby enhances tumor growth and radioresistance in GBM cells. *In vivo* experiments showed that HSP90B1 knockdown combined with radiotherapy can improve the survival rate of mice with GBM (48). Furthermore, natural products from plants and fungi, such as quercetin, can specifically block the function of GLUT-1 transporters. Both *in vivo* and *in vitro* experiments have confirmed that quercetin can significantly inhibit the growth of GBM and extend the survival time of mice (49). These results indicate that quercetin is a potential therapeutic drug for GBM.

Similar to GLUT-1, GLUT-3, also known as the neuronal glucose transporter, is highly expressed in brain tumor-initiating cells (BTICs) and exhibits a higher affinity for glucose (44). The expression of GLUT-3 is significantly higher in Grade 3 and 4 gliomas compared to lower-grade gliomas, suggesting its crucial role in glucose transport in high-grade gliomas (50). Libby et al. further demonstrated that overexpression of GLUT-3 in GBM is associated with increased invasiveness (51). Indeed, experiments have shown that knocking down GLUT-3 can more effectively regulate anaerobic pyruvate utilization and significantly slow down cancer cell proliferation (52). GLUTs enhance cancer cell glucose uptake, driving glycolytic metabolism and providing the necessary energy support for cancer cell survival, proliferation, and growth. Therefore, targeting GLUT1/GLUT3 is considered an ideal strategy to delay GBM tumor cell proliferation and overcome treatment resistance. Kwak S et al. confirmed the efficacy of GBM cell death induction by HDAC2 knockdown, targeting GLUT3 expression, using an *in vivo* orthotopic xenograft tumor model (53). Pucci G et al. demonstrated that GLUT-3 gene knockdown provides a better opportunity to control anaerobic pyruvate utilization and significantly reduces GBM tumor proliferation, suggesting GLUT-3 as a suitable silencing target for overcoming radiation resistance (52).

#### 3.1.2 Monocarboxylate transporters

Excessive lactate generated during glycolysis accumulates, leading to the accumulation of MCTs. MCTs utilize a proton symport mechanism to extrude lactate from cells, which is crucial for maintaining high glycolytic rates in cancer cells and promoting extracellular TME acidification (54–56). Among MCTs, MCT1 is the primary plasma membrane transporter responsible for lactate efflux, particularly in Isocitrate Dehydrogenase (IDH)-wildtype gliomas (57). Consequently, MCT1 is considered a reliable target for targeting glycolytic metabolic reprogramming. Miranda-Gonçalves V et al. showed that inhibiting MCT1 significantly improved the survival rate of brain tumor-bearing mice, reduced lactate efflux and cell invasiveness, and enhanced the survival rate of mice treated with orthotopic gliomas, especially when combined with TMZ therapy, MCT1 knockdown significantly increased the sensitivity of GBM cells to TMZ, thereby significantly prolonging the survival of treated mice (58). Based on these findings, MCT1 inhibitors like AZD3965 have entered Phase I/II clinical trials for the treatment of small cell lung cancer (59) and lymphoma (60).

### 3.2 Targeting key enzymes of glycolytic metabolic reprogramming

#### 3.2.1 Hexokinase 2

HK is a key enzyme in the first step of glycolysis, catalyzing the conversion of glucose to Glucose-6-Phosphate (G-6-P). HK2 is the most well-characterized isoform of the HK family and is significantly overexpressed in GBM tumor cells compared to adjacent normal tissues, correlating with GBM prognosis (61). Wolf A et al. demonstrated that transient inhibition of HK2 in GBM cells suppresses tumor cell proliferation and enhances sensitivity to apoptosis-inducing stimuli such as radiotherapy and chemotherapeutic alkylating agent TMZ. Furthermore, stable HK2 depletion inhibits aerobic glycolysis and promotes normal oxidative glucose metabolism, including decreased extracellular lactate, increased OXPHOS protein expression, and increased oxygen consumption. Interestingly, they found that supplementing HK1 in HK2-deficient cells restored overall HK activity but failed to rescue the aerobic glycolysis phenotype, strongly indicating the unique role of HK2 in GBM progression beyond that of HK1 (62). The role of HK2 in inhibiting tumor cell growth has been confirmed in breast cancer (63), oral cancer (64), and cervical cancer (65). The main therapeutic approaches targeting HK2 include reducing its catalytic activity by inhibiting its expression, directly inhibiting its enzymatic activity to block glucose phosphorylation, and disrupting its interaction with mitochondria to cut off an important energy pathway for tumor cells. Natural products like resveratrol and ginsenosides have been shown to effectively inhibit HK2 activity and exhibit potential in suppressing glycolysis and inducing apoptosis in various cancer experimental models (66, 67). Resveratrol has been shown to significantly inhibit the proliferation of U87 glioma cells and multiple patient-derived GSC cell lines in GBM (68). Additionally, antifungal azole drugs have been identified as a class of potential tumor metabolism inhibitors. Studies have revealed that ketoconazole and posaconazole can effectively inhibit the growth of GBM *in vitro* and *in vivo* by regulating HK2-related genes and signaling pathways. These drugs improve survival rates in GBM mouse models, reduce tumor cell proliferation, and decrease overall tumor metabolism levels (69).

The development of small-molecule HK2 inhibitors is also actively pursued and shows promising prospects. 2-DG is a small-molecule HK2 inhibitor that competitively inhibits HK2-mediated glucose phosphorylation, weakens the immunosuppressive network and macrophage polarization, enhances anti-tumor immunity, and contributes to local tumor control in mouse models (69). The research by Pająk et al. confirms that novel 2-DG analogs, such as WP1122, and HDAC inhibitors (like sodium butyrate and sodium valproate) exhibit significant synergistic anticancer effects, inhibiting the proliferation of GBM cells and inducing apoptosis. *In vivo* experimental results demonstrate that, in mouse models, the combination therapy significantly suppressed tumor growth, improved the survival rate of mice, and no significant toxicity was observed (70). This suggests that 2-DG, as a glycolysis inhibitor, can enhance the sensitivity of GBM cells to radiotherapy and other chemotherapeutic drugs, providing a new strategy for the treatment of GBM. In addition to combination with chemotherapy and radiotherapy, the combination of HK inhibitors and anti-PD-1 antibodies has also shown promising efficacy. HK2 can act as a protein kinase and phosphorylate IκBα at T291, leading to NF-κB-dependent upregulation of PD-L1 expression, which suppresses CD8 T cell activation and infiltration into tumor tissues, accelerating brain tumor growth and increasing resistance to immune checkpoint blockade therapy. Therefore, the combination of HK inhibitors and anti-PD-1 antibodies exhibits superior anti-tumor effects compared to monotherapy with either drug (71).

It is noteworthy that disrupting the interaction between HK2 and mitochondria is another promising strategy. Li R et al. found that Gomisin J, a lignan derivative, can inhibit the proliferation of glioma cells, induce apoptosis, and suppress glycolysis regulated by HK2, thereby inhibiting the progression of gliomas. Gomisin J may represent a potential therapeutic strategy for the treatment of gliomas with relatively low toxicity (72). Similarly, Shteinfer-Kuzmine A et al. focused on cell-penetrating peptides based on Voltage-Dependent Anion Channel 1 (VDAC1), which can perturb the energy and metabolic homeostasis of GBM cells, leading to apoptosis of GBM cells and derived stem cell lines. These peptides also demonstrated inhibitory

effects on tumor growth, invasion, metabolism, stemness, and promoted apoptosis in subcutaneous and intracranial orthotopic GBM xenograft mouse models, suggesting that peptide compounds based on VDAC1 may become a novel candidate for GBM treatment (73). Additionally, the use of VDAC1-specific short interfering RNA (si-VDAC1) to treat GBM cell lines and subcutaneous or intracranial orthotopic GBM xenograft mouse models has been shown to significantly inhibit tumor growth. The remaining tumor tissue exhibited reversed oncogenic characteristics, including metabolic reprogramming, proliferation inhibition, reduced angiogenesis, weakened EMT, decreased invasiveness, and loss of stemness, with some tumor cells differentiating into neuron-like and astrocyte-like cells (74).

#### 3.2.2 Phosphofructokinase-2/fructose-2,6-bisphosphatase 3

Phosphofructokinase-1 (PFK1) is a key regulatory and rate-limiting step in glycolysis, catalyzing the conversion of fructose-6-phosphate and ATP to fructose-1,6-bisphosphate (F-1,6-BP) and ADP (75). PFKFB3 is one of the isoforms of PFK-2 and has been confirmed to be the most highly expressed enzyme in GBM (76). Inhibition of PFKFB3 expression or activity can decrease the level of F-2,6-BP, thereby inhibiting the activity of PFK1 and the rate of glycolysis, ultimately suppressing cell proliferation (77, 78). Clem BF et al. demonstrated that chemical inhibitors targeting PFKFB3, a key enzyme in glycolysis, can effectively reduce glycolysis rate and inhibit tumor cell proliferation (79). This key finding not only confirms the crucial role of PFKFB3 in tumor metabolism regulation but also opens up new strategies for cancer treatment. Li FL et al. further demonstrated the impact of PFKFB3 acetylation status on its activity, showing that acetylated PFKFB3 can significantly promote glycolysis and protect cells from cisplatin-induced apoptosis, revealing the potential role of PFKFB3 modification status in tumor resistance mechanisms (80). Current research trends also lean towards combining PFKFB3 inhibitors with other existing therapies to achieve more comprehensive and effective treatment outcomes. Zhang J’s research team found that simultaneous inhibition of VEGF and glycolysis activator PFKFB3 can significantly prolong the survival of GBM patients and slow down tumor growth. This dual therapy not only inhibited cell proliferation but also promoted apoptosis and enhanced tumor vessel normalization, improving tumor hypoxia, reducing lactate accumulation, and increasing the efficacy and delivery efficiency of chemotherapeutic drugs like doxorubicin (81). This indicates that by integrating the synergistic effects of VEGF and PFKFB3 blockade, it is possible to overcome the current limitations of bevacizumab monotherapy. Nakada M et al. explored the possibility of using PFKFB3 inhibitors to address TKI (tyrosine kinase inhibitor) resistance in GBM, suggesting that the combination of PFKFB3 inhibitors and TKIs may bring breakthroughs in clinical practice (82). Additionally, a series of small-molecule inhibitors targeting PFKFB3 have been extensively investigated, including KAN0438757, developed by Gustafsson NMS et al. Experimental data show that KAN0438757 significantly reduces glioblastoma cell viability and migration ability, with a time- and dose-dependent effect (83). Other inhibitors like 3-PO, PFK15, PFK158, YN1, and N4A have also shown strong antitumor proliferation efficacy in preclinical studies (84). 3-(3-Pyridinyl)-1-(4-pyridinyl)-2-propen-1-one (3-PO) is a novel compound widely reported to inhibit glycolysis flux by competitively inhibiting PFKFB3 (85). Li J’s research team compared the differences in glycolysis flux (analyzed by glucose uptake and lactate secretion) and cell viability (assessed by CCK8 assay) between experimental groups with and without 3-PO addition, providing strong evidence for 3-PO’s role in significantly inhibiting tumor cell glycolytic activity and proliferation (86). The combined application of 3-PO and bevacizumab (a monoclonal antibody targeting VEGF) has been confirmed to reduce GBM cell proliferation and increase apoptosis, thereby delaying tumor growth and improving patient survival (81). PFK15, as a derivative of 3-PO, has demonstrated approximately 100 times higher PFKFB3 inhibitory activity than 3-PO in preclinical studies. Although its anti-GBM growth activity is slightly inferior to that of TMZ, preclinical studies still indicate that PFK15 and its closely related structural derivatives have the potential to exhibit potent cytotoxic activity against various human cancers in future clinical trials. However, it is noteworthy that these compounds have not yet entered the clinical trial phase (79). The combination of PFK158 with CTLA-4 antibody has demonstrated synergistic inhibition of cancer growth, providing a promising perspective for the integration of immunotherapy and targeted glycolytic metabolism therapy (88).

#### 3.2.3 Pyruvate kinase M2

Pyruvate kinase (PK) has two main isoforms: PKM1 and PKM2. It catalyzes the transfer of inorganic phosphate from Phosphoenolpyruvate (PEP) to ADP, generating pyruvate and the energy-rich molecule ATP (82). PKM2 is minimally expressed in healthy brains but highly expressed in GBM cells. Studies have shown that GBM cells undergo a switch from PKM1 to PKM2 isoforms (87). Further experiments have revealed that replacing PKM2 with PKM1 variants in GBM cells leads to restricted biosynthetic pathways and inhibits tumor growth (88). These studies collectively indicate that PKM2 is an important biomarker of glycolytic reprogramming. PKM2 primarily exists in the forms of tetramers and dimers. Dimeric PKM2 is predominantly located in the nuclei of tumor cells and plays a pivotal role in tumor cell proliferation, invasion, and metastasis. Specifically, when the expression of dimeric PKM2 is preferred, it can regulate aerobic glycolysis through metabolic reprogramming of cancer cell metabolic pathways, leading to increased macromolecular biosynthesis in cancer cells and accelerated cancer cell growth. Conversely, activating PKM2 can promote the formation of tetramers, which can inhibit the nuclear import of PKM2 and subsequently suppress the STAT3 signaling pathway, thereby inhibiting the proliferation and metastasis of GBM cells (88, 89). This dynamic equilibrium mechanism between the dimeric and tetrameric forms of PKM2 enables proliferating cancer cells to regulate their anabolic and catabolic demands (90). Liang J et al. demonstrated that activation of the epidermal growth factor receptor (EGFR) induces PKM2 nuclear translocation, upregulating cyclin D1 expression in GBM U87 cell lines and other human cancer cells, accelerating tumor formation (91). On the other hand, the naphthoquinone compound Shikonin, extracted from the traditional Chinese herb Zicao, has been found to inhibit PKM2 activity. *In vitro* experiments have demonstrated its inhibitory effect on the proliferation, migration, and invasion of glioblastoma cell lines U87 and U251. Furthermore, studies have shown that Shikonin can also inhibit the migration and invasion of GBM cells by targeting phosphorylated β-Catenin and phosphorylated PI3K/Akt signaling pathways (92). Ding Y et al. extensively investigated the effects of a series of parthenolide dimers as PKM2 activators and found that compound 5 exhibited high activity. It promotes PKM2 tetramer formation in GBM cells, reduces PKM2 nuclear translocation without affecting total PKM2 expression, and thereby inhibits the STAT3 signaling pathway *in vitro* and *in vivo*, as a result, it suppresses the proliferation and metastasis of GBM cells and induces apoptosis (93). These findings provide potential drug targets and therapeutic approaches for the development of new GBM treatment strategies. Metformin and vitamin K, as PKM2 inhibitors, have shown inhibitory effects on glycolysis, cell proliferation, and drug resistance in various cancers such as osteosarcoma (94), non-small cell lung cancer (95), and hepatocellular carcinoma (96). Metformin has been evaluated for its potential therapeutic effect on glioblastoma in several clinical trials. For example, a cohort study by Adeberg et al. involving 276 patients with primary GBM suggested that the use of metformin in diabetic GBM patients may help slow disease progression and improve patient survival (97),This study provides evidence for metformin as a potential therapeutic agent and lays the foundation for future clinical trials. Additionally, research by Qiao X et al. suggests that metformin can penetrate the blood-brain barrier (BBB) and enter brain tissue, inhibiting the malignant progression of gliomas. The study showed that metformin alone can induce apoptosis in glioma cells (98). However, the specific role of vitamin K in gliomas has not been reported as of now, and further research is needed to elucidate it in the future. Notably, a small molecule compound called dimethylaminochlorocycline (DMAMCL) can modulate glycolysis pathways by activating PKM2, reducing GBM cell proliferation. DMAMCL has been applied in clinical trials for recurrent GBM. This compound selectively binds to the monomeric form of PKM2, promoting PKM2 tetramerization and increasing its pyruvate kinase activity. The inhibitory effect of DMAMCL in GBM cells is weakened when PKM2 is depleted, further demonstrating the critical role of PKM2 in mediating the sensitivity of GBM cells to DMAMCL (99). Furthermore, research by Gao M et al. has shown that Trametinib inhibits the growth and aerobic glycolysis of glioma cells by targeting the PKM2/c-Myc axis. Trametinib suppresses the expression of PKM2 in glioma cells and inhibits the translocation of PKM2 into the nucleus, thereby affecting the expression of c-Myc (100). These findings not only reveal the potential mechanism of Trametinib in antitumor activity but also provide new insights into the clinical drug treatment of gliomas.

#### 3.2.4 Lactate dehydrogenase

Lactate dehydrogenase (LDH) is a core component of the Warburg effect. In hypoxic or oxidative stress conditions, tumor cells adaptively upregulate LDH activity to promote the rapid conversion of pyruvate to lactate during glycolysis, rather than entering the tricarboxylic acid cycle (TCA cycle) for complete oxidation (101, 102). In GBM, the Lactate Dehydrogenase A (LDHA) isoform is typically overexpressed, promoting the flux of substrates towards lactate production during glycolysis, enhancing tumor cell survival and proliferation (103). Studies on various cell lines have shown that inhibiting LDHA function leads to decreased cancer cell proliferation, increased apoptosis, and weakened migration and invasion, further confirming the importance of LDHA as a potential therapeutic target. Galloflavin, a synthesized LDHA and LDHB inhibitor, exhibits high levels of cell penetration (104, 105). Research has shown that Galloflavin inhibits the conversion of pyruvate to lactate, directing pyruvate into the TCA cycle, prompting tumor cells that rely on glycolysis to switch to oxidative phosphoryation (OXPHOS) for energy production, this metabolic shift is detrimental to tumor cells as it increases oxygen consumption and reactive oxygen species (ROS) generation, leading to mitochondrial oxidative damage (106). Gossypol, a derivative of cottonseed oil, has been found to inhibit LDHA. Research by T. Coyle et al. has demonstrated that gossypol exhibits significant *in vitro* and *in vivo* cytotoxicity against central nervous system tumor cells. *In vitro*, gossypol displays concentration- and time-dependent cytotoxicity against various glioma cell lines. *In vivo*, gossypol significantly inhibits tumor growth in a xenograft model in nude mice, suggesting its potential as a therapeutic agent for the treatment of primary malignancies of the central nervous system (107). Gossypol has been shown to be well-tolerated in clinical trials and has shown promise in trials for recurrent malignant gliomas (NCT00540722 and NCT00390403). FX11, a novel gossypol-derived selective LDHA small-molecule inhibitor, has been developed and has shown promise in preclinical studies. It inhibits LDHA activity, decreases intracellular ATP levels, and induces oxidative stress and cell death, which can be partially reversed by the antioxidant N-acetylcysteine (108). Recently, FX11 has been commercialized, and studies in human lymphoma and pancreatic cancer xenografts have shown that it inhibits tumor progression and induces significant oxidative stress and necrosis (108) Daniele S et al. found that NHI-1 and NHI-2 (LDH-A inhibitors) can reduce GBM cell proliferation, trigger apoptosis, and block the cell cycle (103). Additionally, oxalate has been considered to have LDH-inhibitory effects (109). The study of SunT et al. confirmed that Oxamate can reprogram glucose metabolism of cancer stem cells, and can also alleviate immunosuppression of TME and reduce tumor-infiltrating CAR-Treg cells, which may be a potential strategy to enhance CAR-T function in glioblastoma therapy (110). Moorhouse AD et al. designed a novel dual-functional ligand that inhibits LDHA, with activity 9 times higher than sodium oxalate,however, subsequent reports on detailed molecular modeling studies and biological tests targeting cancer cell lines have not been forthcoming (111). The specific application of targeting glycolytic metabolic reprogramming pathways in GBM is demonstrated in **Table 1**.

**Table 1:** Targeted glycolysis inhibitors in the treatment of GBM.

Target	Representative Drugs	Experimental Models	Study Outcomes
GLUT1	HSP90B1	*In vivo*: Intracranial injection of U87, U87-RR (radioresistant U87 cells), and U87-RR/shHSP90B1 (HSP90B1-knockdown U87-RR cells) expressing luciferase. *In vitro*: Three GBM cell lines, namely U251, U87, and LN18, along with human embryonic kidney 293T cells and glioma stem cells (GSCs) MGG8.	*In vitro*: The study found that HSP90B1 is significantly upregulated in radioresistant GBM cell lines. HSP90B1 promotes the localization of GLUT1 on the plasma membrane, enhances glycolytic activity, and subsequently augments tumor growth and radioresistance in GBM cells.*In vivo* effects: In mouse models, HSP90B1 knockdown combined with radiotherapy (IR) improves survival rates in mice harboring GBM (48)
GLUT1	Quercetin	*In vitro*: Human glioblastoma cell lines, U87MG and T98. *In vivo*: Mouse xenograft models were established by subcutaneous injection of U87 cells, and orthotopic GBM models were established by stereotactic intracranial injection of U87 cells.	*In vitro*: **Quercetin significantly inhibited the proliferation ability of GBM cells** *In vivo*:**Q**uercetin significantly inhibited the growth of GBM *in vivo* and prolonged the survival rate of mice (49).
HK2	Ketoconazole and Posaconazole	*In vivo*: Glioblastoma stem cells (GSCs), specifically the cell lines GSC 8–18 and GSC7–2, were injected intracranially. *In vitro*: U87 cells, as well as patient-derived glioblastoma stem cells (GSCs).	*In vitro*: Ketoconazole and posaconazole demonstrated significant inhibitory effects on GBM cell lines and GSCs *in vitro*, with minimal impact on normal cells. *In vivo*: In mouse models, treatment with ketoconazole and posaconazole significantly increased survival rates, reduced tumor cell proliferation, and decreased tumor metabolism. Additionally, treatment with these two drugs increased the proportion of apoptotic cells (69).
HK2	2-DG :WP1122	*In vitro*: Glioblastoma cell lines, U87 and U251 cells. *In vivo*: U87MG cells, intracranial injection.	*In vitro*: The combined use of glycolysis inhibitor WP1122 and HDAC inhibitors (such as sodium butyrate and sodium valproate) demonstrated significant synergistic anti-cancer effects, inhibiting the proliferation of GBM cells and inducing apoptosis. *In vivo*: In mouse models, the combination therapy significantly inhibited tumor growth, improved the survival rate of the mice, and no obvious toxicity was observed (70).
HK2	Gomisin J	*In vitro*: Human glioma cell lines, U87 and U251 *In vivo*: A xenograft mouse model of tumor was established by subcutaneous injection of U251 cells.	• *In vitro*: GomJ significantly inhibited the proliferation of U87 and U251 glioma cells and induced mitochondrial apoptosis, evidenced by the marked upregulation of cytosolic Cyto-c and cleaved Caspase-3. Additionally, GomJ significantly decreased the expression of HK2 in glioma cells and reduced the binding of HK2 to the mitochondrial VDAC, leading to glycolysis inhibition. • *In vivo*: In mouse tumor tissues treated with GomJ, the expression of HK2 was reduced, resulting in a significant decrease in tumor growth, with notably smaller tumor volumes and weights. Furthermore, GomJ exhibited no apparent toxicity to major organs (72).
PFKFB3	KAN0438757	*In vivo*: Human glioblastoma cells, U373 and U251 cell lines.	KAN0438757 significantly reduced the cell viability of U373 and U251 cell lines in a dose-dependent manner. Additionally, KAN0438757 inhibited the migration ability of U373 and U251 cells, showing both time- and dose-dependency (83).
PFKFB3	3-PO	*In vivo*: Patient-derived orthotopic GBM xenograft model, intracranial implantation for inoculation	Combined with bevacizumab reduces GBM cell proliferation and increases apoptosis, thereby delaying tumor growth and improving patient survival (81)
PKM2	Shikonin	*In vitro*: Human glioblastoma cells: U87 and U251 cell lines	Shikonin demonstrates inhibitory effects on the proliferation, migration, and invasion of U87 and U251 cells *in vitro*. Additionally, Shikonin can inhibit the migration and invasion of GBM cells by targeting phosphorylated β-Catenin and phosphorylated PI3K/Akt signaling pathways (92)
PKM2	Parthenolide Dimers	*In vitro*: Human glioblastoma cell lines, U87MG and U251 cell lines. *In vivo*: U87MG cell line, Intracranial injection	*In vitro*: The parthenolide dimer significantly activates PKM2, promotes its tetrameric formation, and inhibits the proliferation and migration of GBM cells. *In vivo*: In mouse models, the parthenolide dimer significantly inhibits tumor growth and improves the survival rate of the mice (93)
PKM2	Metformin	Clinical Trial: Patients with Diabetic GBM	The use of metformin is associated with better overall survival (OS) and progression-free survival (PFS) in glioblastoma (GBM) patients with diabetes. Additionally, the study also explored the impact of metformin on non-diabetic GBM patients and found that its survival-improving effect was more pronounced in diabetic patients (97).
PKM2	DMAMCL	*In vitro*: Human malignant glioma cell lines, including UU118MG, U251MG, SF126, SHG44, and U87MG	By activating PKM2 to regulate the glycolysis pathway, the proliferation of GBM cells is inhibited (99).
PKM2	Trametinib	*In vitro*: Human glioma cell lines, U251 and U87. *In vivo*: Human glioma cell lines, U87 and U251, administered via subcutaneous injection.	*In vitro*: Trametinib inhibited the proliferation, migration, and invasion capabilities of U87 and U251 cells, while inducing apoptosis. Additionally, Trametinib suppressed the expression of glycolysis-related proteins within the cells, including PKM2, GLUT1, and LDHA. *In vivo*: Trametinib inhibited the growth of transplanted tumors *in vivo*. However, overexpression of PKM2 and silencing of c-myc restored the inhibitory effect of Trametinib on the growth of transplanted tumors (100 **).**
LDHA	Gossypol	*In vitro*: Human glioma cell lines include HS 683, U373, U87, and U138, while the mouse glioma cell line is C6. *In vivo*: The BRW cell line (established in the laboratory from a patient with primitive neuroectodermal tumor), subcutaneous injection	Gossypol was administered orally by gavage at a dose of 30 mg/kg per day for four weeks, and it was found that the average tumor weight of the treated xenografts was reduced by more than 50% compared to the untreated xenografts (107).
LDHA	NHI-1 and NHI-2	*In vitro*: U87MG cell line and glioblastoma stem cells (GSCs)	Reduce GBM cell proliferation, trigger apoptosis, and block the cell cycle (103)

## 4 Current challenges and the future perspectives

Glycolytic metabolic reprogramming plays a pivotal role in the development, invasion, and treatment resistance of GBM. It not only provides essential energy and biosynthetic precursors for rapidly proliferating tumor cells but also profoundly shapes the GBM microenvironment, influencing angiogenesis, inflammation, and the dynamic balance of the immune microenvironment. As researchers gain deeper insights into the functions of key glycolytic enzymes and their roles in tumor metabolism regulation, targeting the glycolytic pathway has emerged as a promising therapeutic strategy. Currently, a series of small-molecule inhibitors targeting glycolytic metabolic reprogramming pathways have shown therapeutic effects in preclinical and clinical studies. However, challenges remain, including optimizing drug design to enhance specificity and efficacy, addressing issues related to drug penetration across the blood-brain barrier, potential side effects, and resistance. Combining these inhibitors with existing therapies such as radiotherapy, chemotherapy, targeted therapy, and emerging immunotherapies holds promise for effectively controlling GBM.

In this review, we systematically elucidated the key role of glycolytic metabolic product - lactate, in the occurrence and development of GBM. By thoroughly analyzing existing literature and delving into empirical research, we revealed that abnormal activation of glycolysis is a core feature of GBM metabolic reprogramming, not only providing necessary energy and biomass for the rapid proliferation of tumor cells but also significantly shaping the TME, conferring GBM with enhanced invasiveness and resistance.

Given the central role of glycolytic metabolic reprogramming in GBM, we propose several directions for future research and clinical treatment. Firstly, further elucidation of the molecular mechanisms of glycolysis and their differences in various stages and subtypes of GBM will aid in the development of more precise diagnostic tools and prognostic indicators. For example, monitoring the expression levels, activity, and metabolic product content of key glycolytic enzymes in blood or tumor tissue may serve as powerful biomarkers for assessing disease progression, predicting treatment responses, and prognosis. Secondly, the design and optimization of targeted therapeutic strategies against the glycolytic pathway are crucial. Existing research has shown the potential of drugs inhibiting glycolytic enzyme activity in GBM treatment. However, challenges remain in addressing issues related to drug delivery across the blood-brain barrier, potential side effects, and resistance. Combining these inhibitors with radiotherapy, chemotherapy, and other emerging immunotherapies holds promise for achieving more effective and sustained control of GBM. Finally, given the role of glycolytic products in regulating the TME and immune evasion, future research should focus on the specific effects of glycolytic metabolism on the tumor immune microenvironment, seeking and verifying strategies that can enhance anti-tumor immune responses by regulating glycolysis and altering TME pH. For instance, targeting lactate transporters or interfering with lactate metabolism may help improve immune cell function, enabling better recognition and attack of cancer cells and ultimately enhancing the efficacy of immunotherapy.

## 5 Conclusion

Gaining a deeper insight into and implementing targeted interventions for abnormal glycolysis in GBM offers a promising new strategy to combat this difficult-to-treat tumor. Future research endeavors should concentrate on adapting these fundamental discoveries into clinical applications, thereby furnishing GBM patients with more potent and tailored treatment options.
